# Quality of life and urolithiasis: the patient - reported outcomes measurement information system (PROMIS)

**DOI:** 10.1590/S1677-5538.IBJU.2016.0649

**Published:** 2017

**Authors:** Nishant Patel, Robert D. Brown, Carl Sarkissian, Shubha De, Manoj Monga

**Affiliations:** 1 Department of Urology, Glickman Urological and Kidney Institute, Cleveland Clinic Foundation, Cleveland, OH, USA

**Keywords:** Kidney Calculi, Quality of Life, Pain

## Abstract

**Background:**

With a high rate of recurrence, urolithiasis is a chronic disease that impacts quality of life. The Patient Reported Outcomes Measurement Information System is an NIH validated questionnaire to assess patient quality of life. We evaluated the impact of urolithiasis on quality of life using the NIH-sponsored PROMIS-43 questionnaire.

**Materials and Methods:**

Patients reporting to the kidney stone clinic were interviewed to collect information on stone history and demographic information and were asked to complete the PROMIS-43 questionnaire. Quality of life scores were analyzed using gender and age matched groups for the general US population. Statistical comparisons were made based on demographic information and patient stone history. Statistical significance was P<0.05.

**Results:**

103 patients completed the survey. 36% of respondents were male, the average age of the group was 52 years old, with 58% primary income earners, and 35% primary caregivers. 7% had never passed a stone or had a procedure while 17% passed 10 or more stones in their lifetime. Overall, pain and physical function were worse in patients with urolithiasis. Primary income earners had better quality of life while primary caregivers and those with other chronic medical conditions were worse. Patients on dietary and medical therapy had better quality of life scores.

**Conclusions:**

Urolithiasis patients subjectively have worse pain and physical function than the general population. The impact of pain on quality of life was greatest in those patients who had more stone episodes, underscoring the importance of preventive measures. Stone prevention measures improve quality of life.

## INTRODUCTION

Urolithiasis is a common disease with an estimated prevalence of 13% in men and 7% in women and a rising incidence (1, 2). Recurrence of stones is a common problem with a recurrence rate of 2-5% every year, and the risk of recurrence increases with each stone episode (3). For many, urolithiasis is a chronic disease and chronic diseases are known to have a significant impact on the quality of life (QoL) of the sufferers (4). In spite of this, only a few studies have examined QoL of patients who suffer from urolithiasis, with all demonstrating a decrease in QoL but each having varying results (5).

Patient Reported Outcomes Measurement Information System (PROMIS) is an NIH sponsored online system of validated questionnaires that can be used to evaluate multiple domains related to QoL (6). Results are reported in comparison to both the general population and age and gender matched controls. To date, PROMIS surveys have not been used to evaluate the QoL in those with urolithiasis.

The objective of this study was to compare self-reported QoL in the domains of anxiety, depression, fatigue, pain, physical function, and sleep disturbance of patients diagnosed with urolithiasis to age and gender matched controls using the PROMIS-43 survey.

## MATERIALS AND METHODS

### 

#### Patient Selection

After Institutional Review Board approval, recurrent and first-time kidney stone formers reporting to our kidney stone clinic were recruited to take part in the study. Eligible patients were at least 18 years old with a previous or current diagnosis of kidney stones. All patients were interviewed on the day of their clinic visit to collect basic demographic information including: age, sex, whether primary income earner or primary caregiver, and presence of other chronic illnesses. We also gathered information regarding each patient's history of kidney stone procedures including number and type (ureteral stent placement, shockwave lithotripsy, ureteroscopy, and percutaneous nephrolithotomy), number of stones passed without surgery (based on history), and current dietary (increase in fluids, decrease in oxalate, increase in citrate, decrease in protein, decrease in salt) and medical treatments (use of allopurinol, thiazide diuretic, or potassium citrate).

#### Quality of Life Survey

Following the office interview, each patient was invited to fill out the PROMIS-43 (Version 1) survey. The PROMIS database is an NIH sponsored program with validated questionnaires designed to assess patient function and quality of life in several domains. The PROMIS-43 survey consists of 43 questions assessing the following categories: anxiety/fear, depression/ sadness, fatigue, pain interference, physical function, and sleep disturbance. Individual results are provided for each patient as a T-score percent with the general population mean set at 50% with a standard deviation of 10%. In addition, patients are matched based on age group and sex. For anxiety/fear, depression/sadness, fatigue, pain interference, and sleep disturbance a higher score indicates worse function or quality of life. For physical functioning, a higher score indicates better physical function.

### Statistical analysis

A z-test was performed on all overall scores from each PROMIS category to look for significant differences between those with urolithiasis and the general population. Subgroup analysis was performed to look for differences in QoL based on status as a primary care giver (PCG) to a dependent, status as a primary income earner (PIE) for a household, other significant chronic medical illnesses (SCMI), number of stones passed, number of procedures, age, and sex. T-test and ANOVA were used to compare QoL between groups of urolithiasis patients. Significance was considered at p <0.05.

## RESULTS

### 

#### Demographics

Of 200 invitations, 103 patients completed both the office interview and PROMIS-43 survey over the course of the one year study period. 74% were recurrent stone formers, while 26% were first-time stone formers. 64% of the respondents were female, the mean age in years was 51.6, 58% were the household primary income earners, 35% were primary caregivers and 55% had other chronic medical illnesses. 24% were both PIE and PCG while 27% were neither PIE or PCG.

Patients passed a mean of 11.5 stones in their lifetime without surgery; however, 7% had never passed a stone or had a procedure. Patients underwent 2.4 procedures during their lifetime with shockwave lithotripsy and ureteroscopy being the most common. 30% of patients had made dietary changes for stone prevention, and of these, the mean number of dietary changes was 1.9. 26% of patients were on medical therapy for stone prevention, with 23%, 2% and 1% being on 1, 2 or 3 medications.

#### Overall

**Figure 1:**
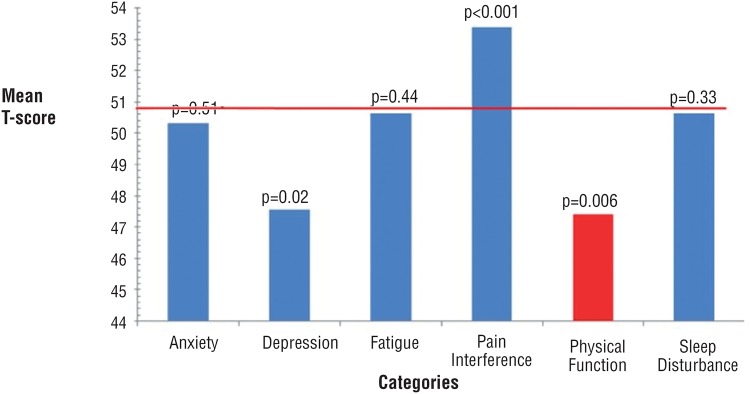
Overall quality of life in patients with urolithiasis. For physical function, a higher T-score indicates better function. For all other categories, a higher T-score indicates worsened quality of life. Population Mean is set at 50% with a standard deviation of 10%.

Overall, reported pain interference and physical functioning were significantly worse in patients with kidney stones compared to the general population. Depression/sadness was significantly less than the general population and there was no statistical difference in anxiety, fatigue or sleep disturbance between the two groups. (Figure-1) **Impact of Demographics on QoL**

**Figure 2:**
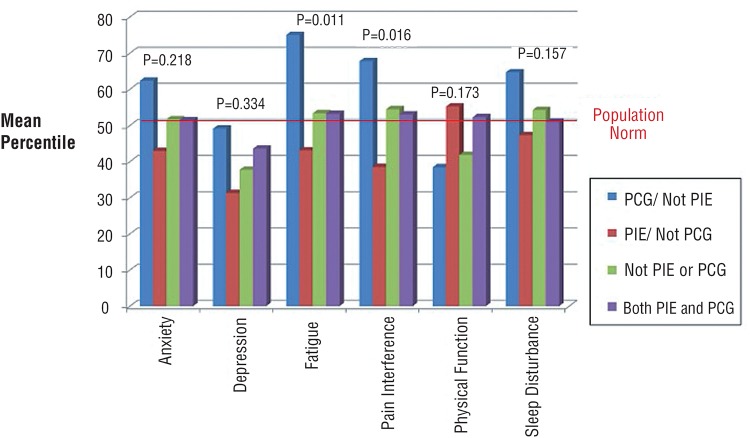
PIE and PCG status. Quality of life based on status as a primary income earner (PIE) and primary caregiver (PCG).

PIE patients had less fatigue, less pain and better physical functioning than those that were not PIE (Table-1). In contrast, stone patients that were PCG had more fatigue and pain than non-PCG patients. When combining PIE and PCG, those that were PIE but not PCG had the best quality of life while those that were PCG but not PIE had the worst quality of life (Figure-2). The presence of co-morbid (SCMI) chronic disease significantly worsened pain and physical functioning. Examining age and gender, those that were under 40 and female had trends showing worse quality of life; however, none of the differences were significant.

**Table 1:** Quality of life based on patient demographics.

		Anxiety		Depression		Fatigue		Pain Interference		Physical Function		Sleep Disturbance	
**Sex**													
	Female	51.01	p=0.28	47.97	p=0.55	52.07	p=0.06	54.63	p=0.09	46.49	p=0.21	50.82	p=0.77
	Male	49.03		46.78		47.92		51.06		49.08		50.31	
**Age**													
	Under 40	53.75	p=0.16	48.10	p=0.85	55.50	p=0.08	55.10	p=0.67	49.1	p=0.47	54.90	p=0.07
	40 to 59	50.86		48.23		50.45		53.18		47.23		49.89	
	60+	48.91		43.27		48.63		52.67		46.36		50.06	
**SCMI**													
	No	50.38	p=0.78	46.27	p=0.18	50.18	p=0.62	51.00	p=0.03	50.93	p<0.01	51.29	p=0.74
	Yes	50.89		48.82		51.25		55.30		44.27		50.73	
**Income**													
	non-PIE/non PCG	50.85		47.81		51.19		54.85		44.59		51.67	
	PCG/non-PIE	54.07	p=0.22	50.36	p=0.33	58.50	p=0.01	58.93	p=0.02	45.00	p=0.17	54.64	p=0.16
	PIE/non-PCG	48.38		45.56		47.56		49.79		49.28		48.87	
	Both PCG and PIE	50.70		48.91		50.35		54.35		48.96		50.00	

Mean T-scores for six PROMIS-43 quality of life measures, p-value is based on T-test measuring in group differences. SCMI (Significant chronic medical illness), PIE (primary income earner), PCG (Primary caregiver)

#### Impact of Stone History on QoL

Patients that passed 10 or more stones and those that had 4 or more procedures in a lifetime had significantly worse pain than those that had fewer stones and procedures (Table-2). Those that made dietary changes had significantly better physical function than those who had not, with other domains trending in the same direction. Patients taking a medication for stone prevention had significantly better depression and anxiety scores.

**Table 2:** Quality of life based on stone history.

		Anxiety		Depression		Fatigue		Pain Interference		Physical Function		Sleep Disturbance	
**Stones**													
	0	50.14		48.06		50.83		52.36		47.25		50.50	
	1 to 2	50.36	p=0.90	47.00	p=0.06	47.96	p=0.28	50.44	p=0.03	49.40	p=0.55	50.28	p=0.34
	3 to 9	49.45		43.73		50.05		53.14		47.50		48.68	
	10+	51.55		51.55		54.20		59.15		45.05		53.50	
**Procedures**													
	0	51.04	p=0.77	48.12	p=0.40	48.64	p=0.25	52.84	p<0.05	49.12	p=0.27	48.64	p=0.39
	1	51.54		49.92		53.19		54.04		44.88		52.12	
	2	49.97		45.79		49.00		50.76		49.21		52.03	
	3	47.25		44.13		49.25		53.50		47.50		47.63	
	4+	49.00		48.70		55.50		61.80		43.40		49.50	
**Diet**													
	No	52.06	p=0.29	49.03	p=0.34	52.68	p=0.24	54.94	p=0.30	44.26	p=0.04	49.94	p=0.41
	Yes	50.03		47.06		49.91		52.67		48.62		51.45	
**Meds**													
	No	51.49	p=0.02	48.87	p=0.02	51.65	p=0.09	52.75	p=0.28	47.30	p=0.86	51.17	p=0.29
	Yes	46.85		43.65		47.58		55.23		47.69		49.08	

Mean T-scores for six PROMIS-43 quality of life measures, p-value is based on t-test or ANOVA comparison within groups. (Stones - number of stones passed over lifetime, Procedures - number of procedures over lifetime, Diet - has the patient made any dietary modifications, Meds - is patient currently on stone prevention medications)

## DISCUSSION

Chronic diseases are known to have an impact on health-related quality of life (4). The NIH sponsored PROMIS database was initially launched in 2004 with the aim to establish a national resource for measurement of patient reported outcomes of health and well-being. Questions were created to measure physical, mental and social health across disease. Several validation studies were performed across multiple diseases by the PROMIS network (6). Further refinement has determined the minimal clinically important differences in PROMIS scores with each domain ranging from 3-6% for the T-score (7). As such, PROMIS questionnaires provide a validated source for determining differences in quality of life across a spectrum of diseases.

Urolithiasis is a common disease with a lifetime prevalence between 10-15% and is increasing in incidence (1, 2). Many of the patients that suffer from urolithiasis will undergo multiple stone episodes with recurrence approaching 100% at 25 years (3). Up to 2 million emergency department visits a year are related to urologic stone disease (5). For many people, urolithiasis is a chronic disease resulting in significant morbidity.

Previous studies have examined the effect of stone episodes on patient psychological well-being. Patients with stressful life factors such as low income, mortgage problems and emotional life events were more likely to have symptomatic stone episodes (8). Miyaoka et al. demonstrated that having multiple stone episodes per year or having symptoms of renal colic were significantly associated with stress (9).

Some studies have linked depression to stone disease. In a prospective study, Angell et al. showed that 30% of stone patients had significant depressive symptoms (10). A large retrospective study in Taiwan found a 1.75 increased risk for a diagnosis of depression in the year following a stone episode (11). There is also a correlation between the frequency and number of stone episodes and anxiety (12).

Other studies have focused on all QoL and these studies have employed the Short Form 36 (SF-36) to evaluate QoL in urolithiasis. While all the studies demonstrate decreased QoL in stone patients, there is little consistency in which health domains are worsened. Diniz et al. showed renal colic was associated with a decrease in QoL in all domains of the SF-36 (13). Bensalah et al. found a decrease in QoL in 5 of 8 domains within the SF- 36, but those with a stone episode within the last month had worse QoL than those with remote episodes (14). Further, other chronic medical illnesses in stone patients significantly worsen QoL (14, 15). Some studies have shown that female stone formers have lower QoL than male counterparts (15, 16). Donnally et al. performed a longitudinal study on QoL with the SF-36 finding that there was no difference in QoL even after improvement in stone symptoms calling into question the validity of the SF-36 (17).

Recently, Penniston and Nakada have developed a new instrument, Wisconsin Stone Quality of Life Questionnaire (WISQOL), to evaluate quality of life in kidney stone patients. Urologists and stone patients identified domains for quality of life that were important in stone disease, and questions were validated on patients presenting to a stone clinic (18). Interestingly, when the WISWOL wad administered to asymptomatic stone formers, a lower QoL was demonstrated in urinary urgency and anxiety (19). The WISQOL performs similarly across multiple sites in regard to health related quality of life (HRQOL) and has thus been internally and externally validated to assess QoL in stone formers (20).

Our results demonstrated an overall decrease in the QoL of stone formers when compared to the general population in two of the six PROMIS-43 categories: pain and physical function. Interestingly, depression scores were lower in the stone forming group. This contrasts with other studies which had shown higher levels of depression within stone formers. The reasons for the differences could be related to the study type. Chung et al. performed a retrospective review that found a correlation between a stone episode and a diagnosis of depression in the following year (11). However, Angell et al. had a prospective study using a validated questionnaire, the Center for Epidemiologic Studies Depression (CES-D) questionnaire, and found an elevation in depression within stone patients (9). Determining the true relationship between depression and urolithiasis will require further study.

A few demographic factors were associated with differences in QoL. Having other chronic medical conditions worsened QoL. Being a household primary income earner meant better QoL while being a primary caregiver worsened QoL. When combining the two, those that were not PIE but were PCG had the worst outcomes. Primary caregivers are known to experience more fatigue and this may impact both self-reported pain and fatigue. In contrast, primary income earners may have been in better overall health to be able to work or may have fewer financial concerns than non-PIE. The reason that non-PIE but PCG has the worst QoL is less clear. It may be that PCG that are not PIE have less financial stability and less support structure than those that are both PIE and PCG. For example, those who work and are primary care givers likely have support at work (sick leave, disability leave) and support at home (daycare, nanny etc.). In contrast, those who are PCG may not have a similar support structure in place. Patients that have other chronic medical illnesses or are primary caregivers may need benefit from more aggressive preventive measures to ward off stone formation, as well as may need consideration for earlier intervention rather than a prolonged course of medical expulsive therapy.

A limitation of our study is an inability to identify where in the continuum of stone disease each patient was at the time of survey. Initially attempting to discern whether stone formers were different from the general population, we treated ‘stone patients’ as a single entity. Knowing kidney stone disease affects QoL scores measured by PROMIS, future studies will help further assess the role of one's clinical course. Considering responses of first time versus recurrent stone formers, acutely symptomatic patients versus those several years removed from a symptomatic episode may further help understanding whether scores are affected by the episodic nature of stone disease.

We could identify an increased burden of disease as measured by number of spontaneously passed stones or number of procedures, negatively impacted pain interference. However, patients who were treated with medical therapy, both dietary and pharmacologic, had improved QoL dimensions. As such, medical therapy should be strongly considered to help improve QoL in all recurrent stone formers.

## CONCLUSION

Urolithiasis negatively impacts quality of life and having more stone episodes worsens quality of life. Certain factors such as chronic medical illnesses and being a primary caregiver for children are associated with lower quality of life. Medical therapy for stones may improve quality of life and should be considered in those with urolithiasis.
